# Lumbar Spine Computed Tomography to Magnetic Resonance Imaging Synthesis Using Generative Adversarial Network: Visual Turing Test

**DOI:** 10.3390/diagnostics12020530

**Published:** 2022-02-18

**Authors:** Ki-Taek Hong, Yongwon Cho, Chang Ho Kang, Kyung-Sik Ahn, Heegon Lee, Joohui Kim, Suk Joo Hong, Baek Hyun Kim, Euddeum Shim

**Affiliations:** 1 Department of Radiology, Korea University College of Medicine, Korea University Anam Hospital, Seoul 02841, Korea; keytech2@naver.com (K.-T.H.); dragon1won@gmail.com (Y.C.); glassesik@gmail.com (K.-S.A.); cielo1462@gmail.com (H.L.); joohee8426@naver.com (J.K.); 2 AI Center, Korea University Anam Hospital, Seoul 02841, Korea; 3 Korea University Guro Hospital, Seoul 02841, Korea; hongsj@korea.ac.kr; 4 Korea University College of Medicine, Korea University Ansan Hospital, Seoul 02841, Korea; kimbaekh@hanmail.net (B.H.K.); edshim1213@gmail.com (E.S.)

**Keywords:** convolution neural network, deep learning, GAN, spine, synthetic image

## Abstract

(1) Introduction: Computed tomography (CT) and magnetic resonance imaging (MRI) play an important role in the diagnosis and evaluation of spinal diseases, especially degenerative spinal diseases. MRI is mainly used to diagnose most spinal diseases because it shows a higher resolution than CT to distinguish lesions of the spinal canals and intervertebral discs. When it is inevitable for CT to be selected instead of MR in evaluating spinal disease, evaluation of spinal disease may be limited. In these cases, it is very helpful to diagnose spinal disease with MR images synthesized with CT images. (2) Objective: To create synthetic lumbar magnetic resonance (MR) images from computed tomography (CT) scans using generative adversarial network (GAN) models and assess how closely the synthetic images resembled the true images using visual Turing tests (VTTs). (3) Material and Methods: Overall, 285 patients aged ≥ 40 years who underwent lumbar CT and MRI were enrolled. Based on axial CT and T2-weighted axial MR images from 285 patients, an image synthesis model using a GAN was trained using three algorithms (unsupervised, semi-supervised, and supervised methods). Furthermore, VTT to determine how similar the synthetic lumbar MR images generated from lumbar CT axial images were to the true lumbar MR axial images were conducted with 59 patients who were not included in the model training. For the VTT, we designed an evaluation form comprising 600 randomly distributed axial images (150 true and 450 synthetic images from unsupervised, semi-supervised, and supervised methods). Four readers judged the authenticity of each image and chose their first- and second-choice candidates for the true image. In addition, for the three models, structural similarities (SSIM) were evaluated and the peak signal to noise ratio (PSNR) was compared among the three methods. (4) Results: The mean accuracy for the selection of true images for all four readers for their first choice was 52.0% (312/600). The accuracies of determining the true image for each reader’s first and first + second choices, respectively, were as follows: reader 1, 51.3% and 78.0%; reader 2, 38.7% and 62.0%, reader 3, 69.3% and 84.0%, and reader 4, 48.7% and 70.7%. In the case of synthetic images chosen as first and second choices, supervised algorithm-derived images were the most often selected (supervised, 118/600 first and 164/600 second; semi-supervised, 90/600 and 144/600; and unsupervised, 80/600 and 114/600). For image quality, the supervised algorithm received the best score (PSNR: 15.987 ± 1.039, SSIM: 0.518 ± 0.042). (5) Conclusion: This was the pilot study to apply GAN to synthesize lumbar spine MR images from CT images and compare training algorithms of the GAN. Based on VTT, the axial MR images synthesized from lumbar CT using GAN were fairly realistic and the supervised training algorithm was found to provide the closest image to true images.

## 1. Introduction

The generative adversarial network (GAN) is a breakthrough deep learning technology that synthesize realistic images that are almost similar to true images. GAN generates new images that did not exist in the past by receiving input of various noises from an artificial neural network and has recently received a lot of attention and has been actively studied. Existing deep learning technology, such as CNN (convolutional neural network), used one multi-layered artificial neural network, but GAN interacts with two artificial neural networks, finally creating a realistic image that is difficult to distinguish. GAN was frequently used to synthesize a new image or change an image, but recently the scope of use has been expanding.

Recent deep learning has allowed its application in medical imaging [1]. The generative adversarial network (GAN) model, which has attracted attention in the field of deep learning, can generate and transform images using two adversarial artificial neural networks, unlike conventional convolutional neural network (CNN) models. GANs can be trained using two adversarial networks, producing realistic images that are almost indistinguishable from real images [2]. The direction of deep learning is opposite to the generative neural network and the discriminative neural network. The generative neural network should make the discriminative neural network think that the realistic image synthesized by the generative neural network is a true image. Conversely, learning should be conducted to determine that the image synthesized by the generative neural network is a fake image by the discriminative neural network.

In medical imaging research, GANs have been used to synthesize positron emission tomography (PET) images from CT images and CT images from MR images [3,4]. In addition, GANs based on unsupervised learning were used to translate CT to MRI images in musculoskeletal images [5].

Computed tomography (CT) and magnetic resonance imaging (MRI) are important for the diagnosis and evaluation of spinal diseases, particularly degenerative spinal diseases. MRI is usually used to diagnose most degenerative spinal diseases because it shows higher resolution than CT in distinguishing lesions of the spinal canals, intervertebral discs, and soft tissues. However, MRI requires significant time for image acquisition, the cost of filming is higher than that of CT, and patients with claustrophobia or MR-incompatible devices sometimes have difficulties with MR examination so that the examination needs careful accommodations, such as requiring special equipment or putting patients in sleep [1]. In addition, if patients cannot afford the cost of MRI, CT is instead used to evaluate spinal disease. In these cases, the evaluation of spinal disease may be limited. However, synthesizing MR images from CT images may allow more accurate and efficient spinal disease diagnosis and evaluation.

Therefore, the purpose of this study was to develop a lumbar spine CT to MRI synthesis AI model using GAN and to validate the performance of realistic synthesis of the model with VTT and qualitive analysis based on GAN.

## 2. Material and Methods

### 2.1. Ethics Statement

This study was approved by the Institutional Review Board and Ethics Committee of Korea University Anam Hospital. The requirement for informed consent was waived because the data were collected retrospectively and analyzed anonymously. The study complied with the ethical principles of the 1964 Declaration of Helsinki revised by the World Medical Organization in Edinburgh in 2000.

### 2.2. Data Preparation for Training and Test

This study enrolled a total of 285 patients aged ≥ 40 years who visited the spine center of the Department of Neurosurgery, Orthopedic Surgery, Rehabilitation Medicine, and Anesthesia Pain Medicine at Korea University Anam Hospital and underwent both lumbar CT and lumbar MRI within 6 months between April 2018 and April 2020. CT scans and MR images were acquired using various models of multidetector CT scanners (IQon Spectral, Philips, Amsterdam, The Netherlands; Ingenuity Core, Philips, Amsterdam, The Netherlands; Somatom Definition Flash, Siemens, Erlangen, Germany; Somatom Definition AS, Siemens, Erlangen, Germany) and 3.0-T MR scanners (Achieva, Philips, Amsterdam, The Netherlands; Magnetom Skyra, Siemens. Erlangen, Germany; Magnetom Prisma Fit; Siemens, Erlangen, Germany).

In the PACS registry, lumbar CT and lumbar MR images satisfying the inclusion criteria for a given period (between April 2018 and April 2020) were obtained. Among the lumbar CT images, images passing through the disc parallel to the vertebral end plate at each level were selected. The lumbar MR images matched with these lumbar CT images were found and stored. The unsupervised and semi-supervised methods started learning with these lumbar CT and MR images. The lumbar CT and MR images were cropped first, and the supervised method started learning with these cropped lumbar CT and MR images. In this way the 285 patients’ data were learned divided into unsupervised, semi-supervised, and supervised methods. For the visual Turing test, 59 additional patients’ data were selected and stored in the way mentioned above. Lumbar MR images were synthesized with the already learned unsupervised, semi-supervised, and supervised methods from the lumbar CT images of 59 patients.

One radiologist with 15 years of experience obtained and reviewed the lumbar CT and MR images for the inclusion criteria in the picture archiving and communication system registry. The inclusion criteria for the study were as follows. First, the dates between CT and MRI did not exceed 6 months. Patients with metallic implants and severe procedures or surgeries that could deform the structure of the lumbar spine were excluded. In most cases, CT was performed for a more accurate evaluation of the bony structure or calcified or ossified lesions after or before the MR examination. Second, patients over 40 years of age were included because our goal was to validate synthetic images in the context of degenerative spinal disease. Third, the patients had no diseases that destroyed the vertebral body or spinal canal, such as spondylitis and malignant tumors; however, patients with mild compression fractures of the vertebral body without spinal canal or disc space involvement were included. Axial CT and T2-weighted MR image data were used. Because this was a preliminary study to confirm the feasibility of the GAN, only one type of MR sequence was selected; namely axial T2-weighted MR images parallel to the endplate of the vertebral body and passing through the middle of the intervertebral disc. CT and MR image pairs with different axes were excluded. A computer scientist (15 years of experience) performed deep learning based on GAN to convert from CT to MR images on the selected dataset.

Cropping of a specific area for the supervised learning of the third algorithm was bounded by the abdominal aorta and IVC at the front, the facet joints at the sides, and the spinous process and paravertebral muscles at the back. The first and second were unsupervised and semi-supervised learning, with lumbar CT totaling 40,173 from L1–2 to the L5–S1 levels and lumbar MRI totaling 9622 from the same level. The third was supervised learning, which is different from the first and second because the image was cropped and matched around the spinal canal at the same vertebra level and the same patient by one radiologist. A total of 4629 lumbar CTs (L1–2: 812, L2–3: 891, L3–4: 1048, L4–5: 1035, and L5–S1: 843), and 3566 lumbar MRIs (L1–2: 558, L2–3: 650, L3–4: 788, L4–5: 800, and L5–S1: 770) were used for supervised learning (Table 1).

### 2.3. Training the GAN to Generate Lumbar MR Images from CT Images

The GAN applied in this study used unsupervised generative attentional networks with adaptive layer-instance normalization (AdaLIN) to translate image (U-GAT-IT) [6], which is an image translation method to create synthetic images. The advantage of this model is that it allows the learning of shape and texture to be learned asymmetrically compared to conventional methods. Loss functions are used, such as adversarial loss, cycle loss, identity loss, and CAM loss. For deep learning, Ubuntu 18.04 was used on a GPU server with three 24 GB memory Titan RTXs, as well as a CUDA toolkit (440.82), and cuDNN 10.2 (NVIDIA Cooperation, Santa Clara, CA, USA). The software environment used for learning was Pytorch 3.xx or higher.

## 3. Deep Learning Framework

**Figure 1:**
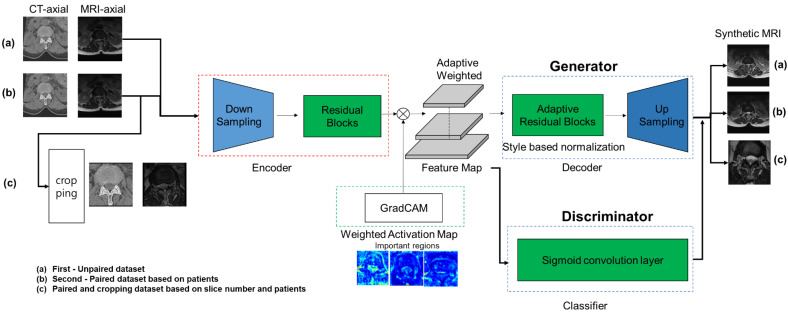
Model architecture for generating synthetic lumbar MRI from real lumbar axial CT images. The detailed explanation is described in the general architecture. MRI, magnetic resonance imaging; CT, computed tomography.

The proposed deep learning architecture for generating synthetic lumbar MRI from real lumbar CT is illustrated in Figure 1. We used the UGAIT [7] integrated attention module to design two generators, $G_{s\to t}$ and $G_{t\to s}$, and two discriminators, $D_{s}$ and $D_{t},$ using lumbar CT and MRI extracted from each domain to convert the real lumbar CT to their corresponding lumbar MRI. The attention module of the generator focuses on specific regions that can be distinguished from other domains. This model was trained by feeding lumbar CT slices with the corresponding real lumbar MRI from each training subject slice by slice (first, unsupervised second, semi-supervised, and supervised learning). Once the deep learning model is trained, it can be used on a new lumbar CT to generate synthetic lumbar MRIs. We customized this framework (UGAIT [7]) to enhance the image generation for synthetic lumbar MRI.

Three different training methods were used to develop the synthesis models in Figure 1. The first (Figure 1a) was an unsupervised learning method that randomly matched lumbar CT and MR images. The synthetic MR images from lumbar CT and the true MR images were randomly compared using this unsupervised method. The second (Figure 1b) was a semi-supervised method that matched lumbar CT and MR images from the same patient. The synthetic and true MR images of the same patient were compared using this method rather than random comparisons. The third (Figure 1c) was the supervised method, in which a specific area was cropped from the same spinal level image of the same patient and then lumbar CT and MR images were matched. At the same level as the lumbar CT image of the same patient, we compared the synthetic MR images in which only a specific part around the vertebral body containing the spinal canal was cropped, to true MR images. Image cropping was performed around the vertebral body and spinal canal. The crop was bounded by the abdominal aorta and inferior vena cava at the front, the facet joints at the sides, and the spinous process and paravertebral muscles at the back.

## 4. General Architecture

**Figure 2:**
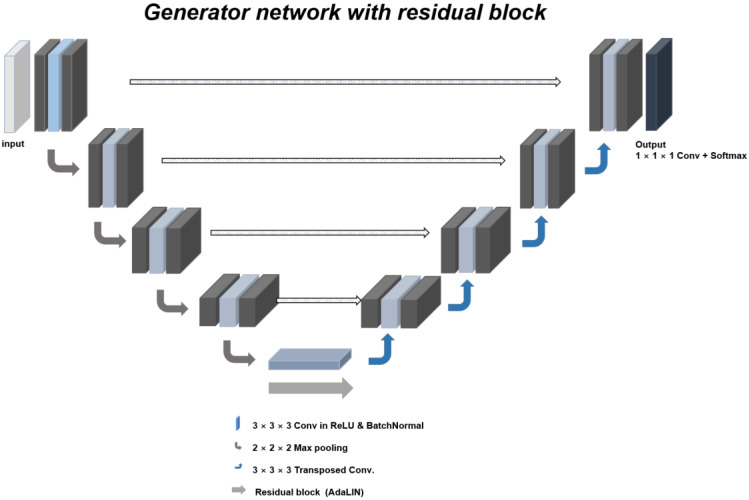
Model architecture of the image generator including residual blocks for upsampling. This network has a ResNet structure. The AdaLin includes fully connected layers and LeakyReLu activation layers. ResNet, residual neural network; AdaLin, adaptive layer-instance normalization; LeakyReLu, leaky rectified linear unit.

For image-to-image translation, we modified UGAIT, which consists of the following steps: a convolution layer, rectified linear unit activation, and instance normalization. The convolution layer included a 3 × 3 kernel, stride-2, and upsampling with the nearest neighbor. In the first step, the number of convolution filters was set to 64 and doubled with every step, reaching 1024 in the last step. Moreover, to concentrate on more important regions and ignore trivial areas for generating images differing between lumbar CT and MRI, this network included the attention map extracted from the auxiliary classifier. These attention maps were integrated into the generator and discriminator to focus on semantically important regions for transforming the shape of the images. While the attention map of the generator induces interesting regions to specifically distinguish between different domains, the attention map of the discriminator can be helpful for fine-tuning to distinguish between real and synthetic images in the target domain. Furthermore, to enhance the style transfer or image translation with different amounts of change in shape and texture, this network consists of AdaLIN by adaptively selecting a proper ratio between layer normalization and instance normalization in residual blocks in Figure 2. However, the disadvantage of this network is that it does not generate regions, such as canals in the spine. To enhance the reconstruction of the synthetic lumbar MR images, we customized the residual block and residual adain block in a single generator. First, the residual block included batch normalization instead of instance normalization and the style-based recalibration module layer for style pooling as a powerful component for image generation. Second, the residual adain block included image processing for blurring at the upsampling bottleneck. We demonstrated these fundamental issues using image translation, which can be learned bidirectionally. This results in many advantages that may address the limitations of the existing cycle GAN or CUT [8,9], as well as U-GAT-IT [7].

**Figure 3:**
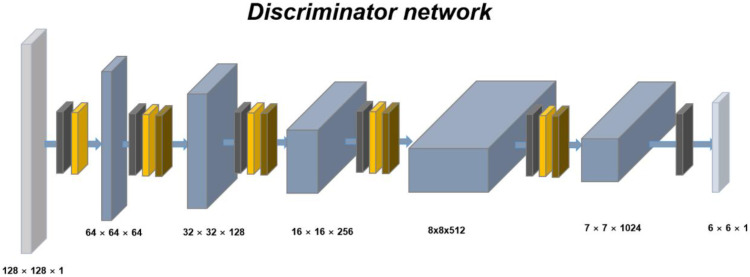
Model architecture of the discriminator for generating synthetic MR images from lumbar spine CT. The generator used a PatchGAN discriminator. Each number of the feature maps is the width, height, and channels of the feature map. The layers for networks were constructed by the color boxes. MR, magnetic resonance; CT, computed tomography.

The discriminator had a structure similar to that of PatchGAN [10]. The architecture of the discriminator is illustrated in Figure 3. The first four convolution layers applied stride-2 and the remaining convolution layers applied stride-1. The first convolution layer inputted 1-channel images and outputted 64-channel feature maps. Subsequently, each time the feature map passed through the convolution layer, the number of channels was doubled. The output was obtained by converting the number of channels to number in the last layer. The discriminator loss, $l_{disc}\left(G,F,D_{x},\;D_{y}\right)$, consisted of the LSGAN losses [11]. Loss Equation (1) was calculated using the output, as follows:

(1) $$l_{disc}\left(G,F,D_{x},\;D_{y}\right)={\;E}_{y\sim_{P_{y\;}}}\left[{\left||D_{Y}\left(y\right)|\right|}_{1}\right]+E_{x\sim_{P_{x\;}}}\left[{\left||1-D_{Y}\left(G\left(x;F\left(c\right)\right)\right)|\right|}_{1}\right]\;\;+{\;E}_{x\sim_{P_{x\;}}}\left[{\left||D_{X}\left(x\right)|\right|}_{1}\right]+{\;E}_{y\sim_{P_{y\;}}}\left[{\left||1-D_{X}\left(G\left(y;C_{x}\right)\right)|\right|}_{1}\right]$$

## 5. Visual Turing Test

**Figure 4:**
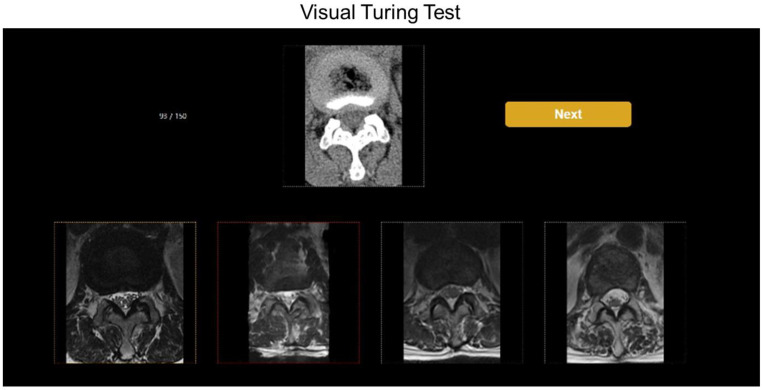
The validation set comprised 150 true and 450 synthetic images developed by 3 algorithms. The true and synthetic images were randomly mixed and displayed on the web solution. Four readers independently determined which MR image best reflected the axial CT image at the disc level and for real. The yellow and red frames of the MR images indicated the first and second choices, respectively. MR, magnetic resonance; CT, computed tomography.

The VTT, which determined how similar the synthetic lumbar MR axial images generated from lumbar CT axial images were to the true lumbar MR axial images, was conducted with 59 patients who were not used in the training data. The method was executed by selecting a set of lumbar MR images composed of one true and three synthetic MR images with reference to the lumbar CT image. For VTT, we designed an evaluation form (Figure 4) comprising 600 axial images (150 true and 450 synthetic images from the unsupervised, semi-supervised, and supervised algorithms) that were randomly distributed.

Two board-certified radiologists (a general radiologist and a musculoskeletal [MSK] radiologist with 15 and 20 years of experience, respectively) and two radiology residents participated in the VTT. We used a program that showed five images (one CT, one true MR, and three synthetic MRI images) on a single screen in random order. The participants were asked to select two MR images that they considered the most accurate among the four lumbar MR images with reference to the CT image (Figure 4). The four radiologists were blinded to each other’s evaluation of the VTT and were not shown the true or synthetic images before the VTT. The number of choices totaled 300, each with a 40-s time limit. The four participants judged the authenticity of each image and chose the first and second candidates for the true image.

The demographics of the 59 patients and the tested spinal levels are shown in Table 1. The VTT excluded 64 levels where CT and MRI were difficult to perform in VTT (8 L1–2, 4 L2–3, 4 L3–4, 13 L4–5, and 35 L5–S1 levels). The reasons for exclusion included mismatching CT and MRI scan directions, which made image comparison difficult, and cases without CT or MRI findings at that level. Finally, 150 CT, 150 true MRI, and 450 synthetic MRI images were selected for the VTT.

## 6. Statistical Analyses

The accuracies of each reader in identifying the true MR image were compared using paired t-tests (R software version 3.5.1; R Foundation for Statistical Computing, Vienna, Austria). The statistical differences in visual comparisons according to each spinal level and the three learning methods were also compared using paired t-tests. Statistical significance was set at *p* < 0.05.

**Figure 5:**
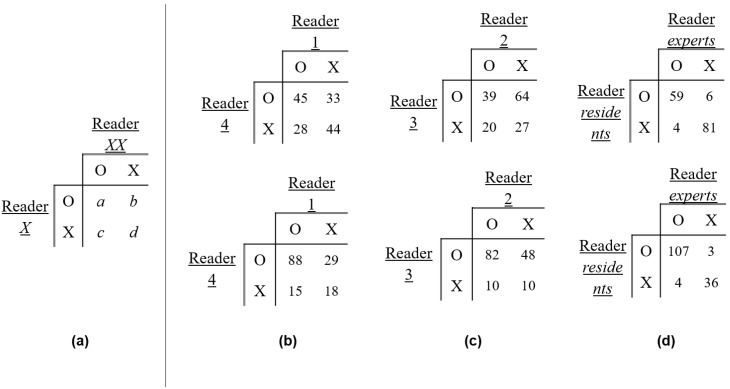
(**a**) An example confusion matrix for identifying real images (**b**) between two expert radiologists; top: priority-first, bottom: priority-first + second, (**c**) between two residents; top: priority-first, bottom: priority-first + second, and (**d**) between experts and resident radiologists; top: priority-first, bottom: priority-first + second.

To analyze the inter-rater reliability for identifying the true images, we calculated the percent positive agreement (PPA) Equation (2), Chamberlain’s percent positive agreement (CPPA) Equation (3) [12,13], and Cohen’s kappa coefficient (K) Equation (4) [14]. These evaluation metrics are commonly used to evaluate the agreement of readers using VTT.

(2) $$\mathrm{PPA}=100\;x\;\frac{2a}{2a+b+c}\;$$

(3) $$\mathrm{CPPA}=100\;x\;\frac{a}{a+b+c}\;$$

(4) $$K=\frac{p_{0}-p_{e}}{1-p_{e}}$$

$$p_{o}=\frac{a+d}{a+b+c+d\;},\;p_{e}=\left(\frac{a+b}{a+b+c+d\;}\times \frac{a+c}{a+b+c+d\;}\right)+\left(\frac{c+d}{a+b+c+d\;}\times \frac{b+d}{a+b+c+d\;}\right)$$

where *a* is the number of cases in which two readers equally found true images, and *b* and *c* are the numbers of cases in which one of the two readers only found true images. *d* indicates the number of cases in which the two readers did not find true images equally. Figure 5a shows an example of a confusion matrix for measuring the PPA, CPPA, and K.

To quantitatively evaluate the three methods of synthetic image quality, Peak SNR (PSNR) and the structural similarity index measurement (SSIM) between the true and synthetic images were used as performance metrics for the developed model as follows:

(5) $$\mathrm{PSNR}\;(x,\;y)=20\log_{10}\frac{MAX_{x}}{||x-{y||}_{2}}$$

(6) $$\mathrm{SSIM}\;(x,\;y)=\frac{\left(2\mu_{x}\mu_{y+C_{1}}\right)\;(2\sigma_{xy}+C_{2)}\;}{\left(\mu_{x^{2}}+\mu_{y^{2}}+C_{1}\right)\left(\sigma_{x^{2}}+\sigma_{y^{2}}+C_{2}\right)}$$

where *C*_1_ = (*K*_1_*L*)^2^ and *C*_2_ = (*K*_2_*L*)^2^. We used *K*_1_ = 0.01 and *K*_2_ = 0.03, as in the original paper [11].

## 7. Results

### 7.1. Accuracy of Identifying the True Images

Regarding the first choice of true images, the mean accuracy for all four readers was 52.0% (312/600). The accuracies of identifying the true images for the first and first + second choices, respectively, for each reader were as follows (Table 2); reader 1, 51.3% (77/150) and 78.0% (117/150); reader 2, 38.7% (58/150) and 62.0% (93/150); reader 3, 69.3% (104/150) and 84.0% (130/150); and reader 4, 48.7% (73/150) and 70.7% (114/150).

### 7.2. Comparisons of Training Methods for Generating Synthetic MR Images

For synthetic images selected as first or second choices, supervised algorithm-derived images were the most frequently selected (118/600 first and 280/600 first + second), followed by semi-supervised (90/600 and 254/600), and unsupervised (80/600 and 220/600) in Table 3. Readers 1 and 3 mainly selected the synthetic lumbar MR images from supervised learning as the true images (reader 1: 38/150 first and 72/150 first + second and reader 3: 32/150 and 92/150). Readers 2 and 4, however, mainly chose synthetic lumbar MR images from unsupervised learning as true images (reader 2: 38/150 first and 77/150 first + second and reader 4: 31/150 and 72/150).

The highest levels of true image selection accuracy among the five spinal levels for readers 1–4 were 66.7% (22/33) for level L3–4, 46.9% (15/32) for level L1–2, 78.8% (26/33) for level L3–4, and 65.6% (21/32) for level L1–2 (Table 2). The mean accuracies of the levels were 53.1% (68/128) for L1–2, 52.3% (69/132) for L2–3, 56.8% (75/132) for L3–4, 50.0% (62/124) for L4–5, and 45.2% (38/841) for level L5–S1. The differences in accuracy between these four readers were not significant (*p* > 0.05).

### 7.3. Evaluations between the Expert and Resident Reader Groups

Our analysis of the inter-rater reliability for identifying true images showed PPA, CPPA, and K (Figure 5 and Table 4) values for the two expert readers of 59.6%, 42.5%, and 0.187 (first), and 80.0%, 66.7%, and 0.258 (first + second), respectively. The values for the two resident readers were 48.15%, 31.7%, −0.389 (first) and 66.7%, 58.6%, 0.072 (first + second), respectively. The PPAs, CPPAs, and Ks for all readers were 92.2%, 85.5%, and 0.845 (first), and 96.8%, 93.9%, and 0.880 (first + second), respectively (Table 4).

### 7.4. Evaluations of PSNR and SSIM among the Three Algorithms

The results for quantitative image quality among the three algorithms (unsupervised, semi-supervised, and supervised training) are shown in Table 5. The PSNRs of each slice among the 59 patients of the test datasets were 15.278 ± 0.830 (unsupervised), 15.319 ± 1.037 (semi-supervised), and 15.987 ± 1.039 (supervised), respectively. The SSIMs of each slice among the 59 patients of the test datasets were 0.490 ± 0.051 (unsupervised), 0.479 ± 0.048 (semi-supervised), and 0.518 ± 0.042 (supervised), respectively.

## 8. Discussion

### 8.1. The Research of Other Algorithms and GAN

GAN is a learning technique that has recently been a focus of deep learning using AI, which is used to generate or transform images using adversarial generative neural networks to create artificial but realistic-looking images [6,15]. While conventional CNN models have utilized a method to train one multilayer artificial neural network, GAN differs in progressing learning by the interaction of two artificial neural networks. In the presence of generative and discriminative neural networks, generative neural networks are trained such that their images can be truly distinguished in discriminative neural networks, and discriminative neural networks are trained to discriminate images made in generative neural networks as fake images. Through the adversarial learning process of these two neural networks, a GAN can generate synthetic images that are difficult to distinguish from real images [3].

Recently, researchers have searched for methods to replace MRI with CT scans when planning radiation therapy [16,17,18]. However, CT-based MRI construction has received little attention. It is challenging to generate an MR image directly from a CT image using a linear model because it is difficult to generate high-level image domains based on low-level images. In response, we proposed a synthesis method based on CNNs [19] with adversarial training [20] to generate a lumbar spine MR image from a CT scan. A 2019 study synthesized MR images from brain CT images using GAN [21], and studies published in 2017 reported the process of converting brain MR images to CT using GAN [22]. In addition, studies have reported on the conversion of images from one modality into images from another using GAN. Lee et al. reported the synthesis of spine MR images from spine CT images using GAN, with a mean overall similarity of synthetic MR T2-weighted images evaluated by radiologists of 80.2% [23]. They concluded that the synthetic MR images from spine CT images using GANs would improve the diagnostic usefulness of CT.

This is a preliminary step in determining whether lumbar synthetic MR images generated from lumbar CTs by applying GAN are clinically applicable. We first assessed whether the synthetic images generated from lumbar CT scans were distinguishable from the true MR images. If radiologists with various experiences find it difficult to distinguish between true and synthetic MRIs via VTT, the MR images synthesized through GAN may be sufficiently similar to true MRIs [24] and warrant testing in the clinical setting. The first study on lumbar spine MR image synthesis from CT was published in 2020 [25]. Using a small dataset, the authors generated synthetic lumbar spine MR images using GAN and determined the similarities between the synthesized and true MR images. In contrast to this work, we did not perform quantitative comparisons using the mean absolute error and peak signal-to-noise ratio or qualitative comparisons of each structure of the spine, including the discs, facet joints, spinal canals, and thecal sacs.

In medical imaging, computer-based vision evaluation methods are largely used to measure detection and segmentation accuracy, emphasizing the classification of regions according to anatomy from a predefined library. As an alternative, motivated by the ability of humans to provide far richer descriptions, we constructed a VTT that used binary questions to probe a model’s ability to distinguish fake images from true images. In our VTT, the probability of finding a true image was 52%; in other words, the ability to distinguish between real and fake images was half of the time. This probability is the same as in the situation in which a coin is thrown to predict which side will land facing upward. The results of VTT indicated that the GAN model developed in this study made synthetic lumbar MR images that were difficult to distinguish from real images.

A previous study applied a VTT to determine how synthetic lung nodules generated by GAN compared to the original lung nodules on CT [24]. Two radiologists participated in the VTT; the authors concluded that it was difficult for radiologists to distinguish between the generated and real nodules. A neuroimaging study also using a VTT [25] generated synthetic brain MR images using GANs and compared them to true brain MR images by VTT by an expert physician looking at 50 synthetic and 50 true MR images in random order and determining whether they were true or synthetic. The authors concluded that it was difficult for the expert physician to accurately distinguish between synthetic and true brain images. Synthetic high-resolution body CT images with progressive growing GAN (PGGAN) were also indistinguishable from real images in VTT [6].

### 8.2. The Present Study for Conversion from CT and MR Images

The present study utilized GAN trained with unsupervised, semi-supervised, and supervised methods and compared their fake image synthesis performance through VTTs. Supervised learning uses aligned training datasets in which the output image corresponds to each input image. By disconnecting the aligned data into an input and output set to train, medical synthesis becomes an unsupervised learning-based synthetic task. A semi-supervised learning can be configured to utilize both supervised and unsupervised learning. A highly supervised training typically requires a large volume of labeled datasets [26]. However, acquiring those from expert radiologists at a sufficient scale can be prohibitive; thus, we anticipated unsupervised training, which meant the unpairing of the CT and MR data, although our results showed that supervised training-derived images were selected most often as the first and second choices. In other words, the images produced using the supervised method were the most realistic images.

In contrast to our results, brain MRI to CT synthesis research showed that unpaired data-derived images were more realistic and contained fewer artifacts and less blurred images in comparisons of the conversion between unpaired and paired data using mean absolute error (MAE) values and peak-signal-to-noise ratio (PSNR) in true and synthesized CT [22]. Another study on transforming brain CT into MRI using GAN reported that the combination of paired and unpaired data showed more realistic images in MAE and PSNR than using paired or unpaired data individually [21]. The authors also reported that this combination solved the context-misalignment problem of unpaired training and alleviated the rigid registration task and blurred results of paired training. First, an unsupervised method of learning by randomly matching lumbar CT and MR images was used to convert CT into MRI; however, the main part of the synthetic MR images was converted differently from the real MR images in some synthetic MR images. To solve this problem of the first method, a learning method was performed with CT and MR image pairs for each patient. Finally, although the image conversion of CT to MR is more difficult than that of MR to CT, the use of paired data and cropped information can be more helpful for generating synthetic MR images. The above two studies were evaluated through measurements such as MAE and PSNR, but we attempted to evaluate the images through a VTT. Therefore, it is difficult to compare our results to those of previous studies. We also observed no difference in the accuracy of separating synthetic from true MRIs and in the inter-reader agreements among four expert and resident radiologists, providing indirect evidence that synthetic MRIs and true MRIs had comparable image fidelity, although the selection criteria between readers were likely to be subjective and differ according to experience.

The results of VTTs according to lumbar spine levels showed the highest rate of true image selection for the L3–4 level (56.8%), followed by the L1–2 level (53.1%). The L5–S1 level showed the lowest rate (45.2%), likely because the anatomical shape that changes from the lumbar spine to the sacrum differs from the other lumbar levels, making it more difficult to determine true or synthetic MR images than other lumbar levels based on the reference CT. However, the choice between lumbar levels did not differ significantly. For image quality, the supervised algorithm received the best score (PSNR: 15.987, SSIM: 0.518 ± 0.042, respectively in Table 5).

### 8.3. The Limitations of Our Study

This study has several limitations. The first is the limitation of the VTT. This test was used to assess how intuitively similar the synthetic MR images were to the true MR images and not to evaluate how well the synthetic MR images replicated the individual structures of true MR images. Our future goal is to determine whether MR images synthesized using GAN from lumbar CT images have clinical significance compared to true MRIs through structural analysis of the disc, spinal canal, and paraspinal muscles of the lumbar spine. The second limitation was that the L5–S1 levels were excluded from the study. In some CT examinations, continuous scans were performed from the superior end plate of L1 through the S1 level without adjusting the horizontal direction of the disc level. Therefore, the axial levels of CT often have different directions from those of MRI, which is strictly scanned around the intervertebral disc, making it difficult to compare images. Therefore, this study excluded 35 cases with L5–S1 images (L1–2: 8 cases, L2–3: 4 cases, L3–4: 4 cases, and L4–5: 13 cases). The third limitation was caused by image cropping in deep learning using the supervised method. In axial lumbar CT and MR images, the same part cannot be cropped around the vertebral body. Fourth, the training dataset of our study was small. We used 285 CT scans in deep learning with GAN, while other studies used 11,755 body CT scans during PGGAN training and 1018 lung cancer screening thoracic CT scans during DC-GAN training. The fourth limitation is related to the low number of patients and different CT scanners. A total of 285 patients’ CT images were used for deep learning and 59 patients’ CT images were used for the VTT that were not included in deep learning. Although five levels of CT images were used per patient, the limitation of this study was that a large number of CT images were not included in the deep learning, and another limitation of this study is that all patients could not be taken with the same type of CT equipment.

## 9. Conclusions

We developed three methods of a GAN model to convert from lumbar CT to MR images, which were evaluated with a VTT. Based on the VTT, the axial MR images synthesized from lumbar CT using GAN were fairly realistic and the supervised training algorithm was found to provide the closest image to true images.

If future research validates the clinical usefulness of replacing true lumbar spine MR images with synthetic images in particular cases, the lumbar spine CT to MR synthesis using GAN could expand the role of CT, which is traditionally narrowed in the diagnosis of degenerative spinal disease, and could also increase the diagnostic value of CT with additional reference information.

## Figures and Tables

**Table 1:** Demographic characteristics of the study population by group.

	Training (with Tuning)		Test (VTT)
Patients	285		59
CT slices	Unsupervised training Semi-supervised training	40,173	150 CT axial images
	Supervised training	4629	
Level-L1–2	812		32
Level-L2–3	891		33
Level-L3–4	1048		33
Level-L4–5	1035		31
Level-L5–S1	843		21
MRI slices	Unsupervised training Semi-supervised training	9622	150 true and 450 synthetic MR axial images
	Supervised training	3566	
Level-L1–2	558		32 + 96
Level-L2–3	650		33 + 99
Level-L3–4	788		33 + 99
Level-L4–5	800		31 + 93
Level-L5–S1	770		21 + 63
Age (years)			
Male	63.18 ± 16.47		68.56 ± 4.24
Female	68.08 ± 15.46		69.66 ± 7.07
Sex			
Male	129		18
Female	156		41

Note: The number of levels was used for training in the third method (matching of level and patients). There were no demographic differences between the training and test groups (*p* = 0.1163 for age and *p* = 0. for sex in the datasets). CT, computed tomography; MRI, magnetic resonance imaging; VTT, visual Turing test.

**Table 2:** Assessment of the choice of true lumbar MR images through the VTT by the four readers.

		Visual Turing Test						*p*-Value
		Total	Level 1–2	Level 2–3	Level 3–4	Level 4–5	Level 5–S1	
Reader 1	first	51.3% (77/150)	40.6% (13/32)	51.5% (17/33)	66.7% (22/33)	58.1% (18/31)	33.3% (7/21)	reference
	first + second	78.0% (117/150)	81.3% (26/32)	75.8% (25/33)	87.9% (29/33)	77.4% (24/31)	61.9% (13/21)	reference
Reader 2	first	38.7% (58/150)	46.9% (15/32)	39.4% (13/33)	42.4% (14/33)	32.3% (10/31)	28.6% (6/21)	0.2497
	first + second	62.0% (93/150)	78.1% (25/32)	60.6% (20/33)	69.7% (23/33)	58.1% (18/31)	41.6% (10/21)	0.2178
Reader 3	first	69.3% (104/150)	59.4% (19/32)	66.7% (22/33)	78.8% (26/33)	67.7% (21/31)	76.2% (16/21)	0.1190
	first + second	84.0% (130/150)	81.3% (26/32)	81.8% (27/33)	87.9% (29/33)	90.3% (28/31)	95.2% (20.21)	0.4396
Reader 4	first	48.7% (73/150)	65.6% (21/32)	51.5% (17/33)	39.4% (13/33)	41.9% (13/31)	42.9% (9/21)	0.8125
	first + second	70.7% (114/150)	81.3% (26/32)	78.8% (26/33)	60.6% (20/33)	67.7% (21/31)	61.9% (13/21)	0.9671
Total	first	52.0% (312/600)	53.1% (68/128)	52.3% (69/132)	56.8% (75/132)	50.0% (62/124)	45.2% (38/84)	-
	first + second	74.3% (446/600)	78.1% (100/128)	74.2% (98/132)	76.5% (101/132)	73.4% (91/124)	66.7% (56/84)	-

Note: Readers 1 and 4 are expert radiologists and readers 2 and 3 are resident radiologists. MR, magnetic resonance; VTT, visual Turing test.

**Table 3:** Comparisons of the selected proportions of the three deep learning algorithms.

		Deep Learning Algorithm		
		Unsupervised	Semi-Supervised	Supervised
Reader 1	first	10	25	38
	first + second	45	66	72
Reader 2	first	38	28	26
	first + second	77	72	58
Reader 3	first	1	13	32
	first + second	26	52	92
Reader 4	first	31	24	22
	first + second	72	64	58
Total	first	80/600 (13.3%)	90/600 (15.0%)	118/600 (19.7%)
	first + second	220/600 (36.7%)	254/600 (42.3%)	280/600 (46.7%)

**Table 4:** Three inter-reader agreements for identifying the true MR images for each reader, including the expert and resident radiologists.

		PPA (%)	CPPA (%)	K
Two expert radiologists	first	59.6	42.5	0.187
	first + second	80.0	66.7	0.258
Two resident radiologists	first	48.2	31.7	−0.389
	first + second	66.1	58.6	0.072
Expert radiologists versus Resident radiologists	first	92.2	85.5	0.845
	first + second	96.8	93.9	0.880

PPA, percent positive agreement; CPPA, Chamberlain’s percent positive agreement; K, Cohen’s kappa coefficient; first, first selection of each reader; second, second selection of each reader.

**Table 5:** Overall statistics for two measures of model quality for three algorithms (unsupervised, semi-supervised, and supervised): PSNR and SSIM. The average and standard deviation for each measure about from axial slices of the 5 spine levels among the 59 subjects in our test datasets.

		PSNR	SSIM
First method: Unsupervised learning	Level 1–2	16.062 ± 1.347	0.538 ± 0.060
	Level 2–3	15.678 ± 1.647	0.526 ± 0.067
	Level 3–4	15.772 ±1.352	0.507 ± 0.062
	Level 4–5	14.844 ± 1.350	0.465 ± 0.068
	Level 5–S1	14.033 ± 1.258	0.412 ± 0.064
	Total	15.278 ± 0.830	0.490 ± 0.051
Second method: Semi-supervised learning	Level 1–2	16.234 ± 1.964	0.529 ± 0.069
	Level 2–3	16.149 ± 2.020	0.515 ± 0.073
	Level 3–4	15.708 ±1.824	0.492 ± 0.069
	Level 4–5	14.670 ± 1.729	0.448 ± 0.075
	Level 5–S1	13.836 ± 1.865	0.398 ± 0.079
	Total	15.319 ± 1.037	0.479 ± 0.048
Second method: Semi-supervised learning	Level 1–2	16.554 ± 1.203	0.557 ± 0.094
	Level 2–3	16.732 ± 1.395	0.553 ± 0.102
	Level 3–4	16.560 ±1.116	0.544 ± 0.084
	Level 4–5	15.863 ± 1.449	0.521 ± 0.087
	Level 5–S1	14.228 ± 1.341	0.455 ± 0.076
	Total	15.987 ± 1.039	0.518 ± 0.042

Note: bold is the best score.

## Data Availability

The datasets used and/or analyzed during the current study are available from the corresponding author upon reasonable request.

## References

[1] Chartrand G., Cheng P.M., Vorontsov E., Drozdzal M., Turcotte S., Pal C.J., Kadoury S., Tang A. (2017). Deep Learning: A Primer for Radiologists. RadioGraphics.

[2] Jo Y.J., Bae K.M., Park J.Y. (2020). Research trends of generative adversarial networks and image generation and translation. Electron. Telecommun. Trends.

[3] Bi L., Kim J., Kumar A., Feng D., Fulham M., Cardoso M.J., Arbel T. (2017). Synthesis of positron emission tomography (PET) images via multi-channel generative adversarial networks (GANs). Molecular Imaging, Reconstruction and Analysis of Moving Body Organs, and Stroke imaging and Treatment.

[4] Nie D., Trullo R., Lian J., Petitjean C., Ruan S., Wang Q., Shen D. (2017). Medical Image Synthesis with Context-Aware Generative Adversarial Networks. International Conference on Medical Image Computing and Computer-Assisted Intervention.

[5] Jin C.-B., Kim H., Liu M., Han I.H., Lee J.I., Lee J.H., Joo S., Park E., Ahn Y.S., Cui X. (2019). DC2Anet: Generating Lumbar Spine MR Images from CT Scan Data Based on Semi-Supervised Learning. Appl. Sci..

[6] Park H.Y., Bae H.-J., Hong G.-S., Kim M., Yun J., Park S., Chung W.J., Kim N. (2021). Realistic High-Resolution Body Computed Tomography Image Synthesis by Using Progressive Growing Generative Adversarial Network: Visual Turing Test. JMIR Med. Inform..

[7] Kim J., Kim M., Kang H., Lee K. (2019). U-gat-it: Unsupervised generative attentional networks with adaptive layer-instance normalization for image-to-image translation. arXiv.

[8] Zhu J.Y., Park T., Isola P., Efros A.A. Unpaired image-to-image translation using cycle-consistent adversarial networks. Proceedings of the 16th IEEE International Conference on Computer Vision.

[9] Park T., Efros A.A., Zhang R., Zhu J.-Y. (2020). Contrastive Learning for Unpaired Image-to-Image Translation. European Conference on Computer Vision.

[10] Isola P., Zhu J.-Y., Zhou T., Efros A.A. Image-to-Image Translation with Conditional Adversarial Networks. Proceedings of the 2017 IEEE Conference on Computer Vision and Pattern Recognition (CVPR).

[11] Wang Z., Bovik A.C., Sheikh H.R., Simoncelli E.P. (2004). Image quality assessment: From error visibility to structural similarity. IEEE Trans. Image Process..

[12] Bartlett J.W., Frost C. (2008). Reliability, repeatability and reproducibility: Analysis of measurement errors in continuous variables. Ultrasound Obstet. Gynecol..

[13] Kong K.A. (2017). Statistical Methods: Reliability Assessment and Method Comparison. Ewha Med. J..

[14] Cohen J. (1960). A Coefficient of Agreement for Nominal Scales. Educ. Psychol. Meas..

[15] Kim T., Cha M., Kim H., Lee J.K., Kim J. (2017). Learning to discover cross-domain relations with generative adversarial networks. International Conference on Machine Learning.

[16] Hsu S.-H., Cao Y., Huang K., Feng M., Balter J.M. (2013). Investigation of a method for generating synthetic CT models from MRI scans of the head and neck for radiation therapy. Phys. Med. Biol..

[17] Zheng W., Kim J.P., Kadbi M., Movsas B., Chetty I.J., Glide-Hurst C.K. (2015). Magnetic Resonance–Based Automatic Air Segmentation for Generation of Synthetic Computed Tomography Scans in the Head Region. Int. J. Radiat. Oncol..

[18] Kapanen M., Tenhunen M. (2013). T1/T2*-weighted MRI provides clinically relevant pseudo-CT density data for the pelvic bones in MRI-only based radiotherapy treatment planning. Acta Oncol..

[19] Krizhevsky A., Sutskever I., Hinton G.E. Imagenet classification with deep convolutional neural networks. Proceedings of the Advances in Neural Information Processing Systems.

[20] Goodfellow I., Pouget-Abadie J., Mirza M., Xu B., Warde-Farley D., Ozair S., Courville A., Bengio Y. Generative adversarial nets. Proceedings of the Advances in Neural Information Processing Systems.

[21] Jin C.-B., Kim H., Liu M., Jung W., Joo S., Park E., Ahn Y.S., Han I.H., Lee J.I., Cui X. (2019). Deep CT to MR Synthesis Using Paired and Unpaired Data. Sensors.

[22] Wolterink J.M., Dinkla A.M., Savenije M.H., Seevinck P.R., van den Berg C.A., Išgum I., Tsaftaris S., Gooya A., Frangi A., Prince J. (2017). Deep MR to CT synthesis using unpaired data. Simulation and Synthesis in Medical Imaging. SASHIMI 2017. Lecture Notes in Computer Science.

[23] Lee J.H., Han I.H., Kim D.H., Yu S., Lee I.S., Song Y.S., Joo S., Jin C.-B., Kim H. (2020). Spine Computed Tomography to Magnetic Resonance Image Synthesis Using Generative Adversarial Networks: A Preliminary Study. J. Korean Neurosurg. Soc..

[24] Chuquicusma M.J.M., Hussein S., Burt J., Bagci U. How to fool radiologists with generative adversarial networks? a visual turing test for lung cancer diagnosis. Proceedings of the IEEE 15th International Symposium on Biomedical Imaging.

[25] Han C., Hayashi H., Rundo L., Araki R., Shimoda W., Muramatsu S., Furukawa Y., Mauri G., Nakayama H. GAN-based synthetic brain MR image generation. Proceedings of the IEEE 15th International Symposium on Biomedical Imaging (ISBI 2018).

[26] Gulshan V., Peng L., Coram M., Stumpe M.C., Wu D., Narayanaswamy A., Venugopalan S., Widner K., Madams T., Cuadros J. (2016). Development and validation of a deep learning algorithm for detection of diabetic retinopathy in retinal fundus photographs. JAMA.

